# CXCL1 as an Unfavorable Prognosis Factor Negatively Regulated by DACH1 in Non-small Cell Lung Cancer

**DOI:** 10.3389/fonc.2019.01515

**Published:** 2020-01-10

**Authors:** Shengnan Yu, Ming Yi, Linping Xu, Shuang Qin, Anping Li, Kongming Wu

**Affiliations:** ^1^Department of Oncology, Tongji Hospital of Tongji Medical College, Huazhong University of Science and Technology, Wuhan, China; ^2^Department of Medical Oncology, The Affiliated Cancer Hospital of Zhengzhou University & Henan Cancer Hospital, Zhengzhou, China

**Keywords:** NSCLC, lung adenocarcinoma, CXCL1, DACH1, progression, prognosis

## Abstract

**Background:** Interaction between cancer cells with microenvironment is essential for cancer progression, therapeutic resistance and prognosis. Chemokine CXCL1 shows variable roles in the development of cancers. DACH1 has been considered as a tumor suppressor and represses the expressions of several chemokines. The relationship between CXCL1 and DACH1 in non-small cell lung cancer (SCLC) deserves further investigation.

**Methods:** Immunohistochemistry staining was performed on tumor tissue microarrays from lung cancer patients to detect CXCL1 protein. The CXCL1 concentration in the serum of adenocarcinoma patients was measured by ELISA. The CXCL1 protein secreted by cancer cell lines was detected by SearchLight proteome array and human cytokine antibody array. The meta-analysis of CXCL1 expression form public databases was performed and correlation between CXCL1 and DACH1 was analyzed. Moreover, the association between clinicopathological features and prognosis with CXCL1 and DACH1 was analyzed by tissue array and KM-plotter from public database.

**Results:** The protein abundance of CXCL1 in lung cancer tissues was significantly higher than that in adjacent normal tissues. CXCL1 was closely related to TNM stage, tumor size, and lymph node metastasis and predicted worse overall survival in adenocarcinoma. The level of CXCL1 in the peripheral blood of adenocarcinoma patients also significantly elevated and positively related with clinical stage. The meta-analysis demonstrated that CXCL1 mRNA level was increased in lung cancer tissues and high level of CXCL1 indicated tumor progression in lung adenocarcinoma. In addition, public database analyses showed that CXCL1 negatively correlated with DACH1. Stable overexpressing DACH1 in cultured lung cancer cells remarkably decreased CXCL1 protein. Moreover, ectopic expression of DACH1 significantly inhibited the expression of CXCL1, Ki67, and cyclin D1 in tumor tissues compared with A549 cells with empty vector. Survival analysis showed that high CXCL1 and low DACH1 indicated poor overall survival and progression-free survival.

**Conclusion:** CXCL1 is closely associated with tumor progression and poor survival. DACH1 significantly inhibits the expression of CXCL1 and indicates good prognosis. Therefore, combined detection of CXCL1 and DACH1 could more precisely predict prognosis of lung adenocarcinoma.

## Background

Lung cancer is the most common cause of cancer death in male and the second leading cause in female all over the word (1). Lung cancer is divided into small cell lung cancer (SCLC) and non-small cell lung cancer (NSCLC). NSCLC includes adenocarcinoma (ADC), squamous cell carcinoma (SQC) and large cell lung cancer, etc. At present, surgery, radiotherapy and chemotherapy, and target therapy are the main therapeutic methods for NSCLC (2), which prolonged patients' survival. However, recurrence and resistance remain an urgent problem (3–5). Epidermal growth factor receptor (EGFR) mutations and anaplastic lymphoma kinase (ALK) -rearrangements are typically associated with tumorigenesis and prognosis of patients (6, 7). However, the mechanism of the oncogenesis and progression of NSCLC still requires more exploration. In a search for lung tumor suppressor by genomic landscape, DACH1 was described as a potential tumor suppressor in non-small cell lung cancer (NSCLC) (8).

Human Dachshund homolog 1 (DACH1) is a key member of retinal determination gene network (RDGN), which regulated cell proliferation, apoptosis, tumor growth and progression (9, 10). In comparison with normal tissues, DACH1 protein and mRNA expression level obviously decreased in several tumor tissues, including lung cancer (8, 11, 12). The reduction of DACH1 was mainly caused by genomic deletion and promoter region hypermethylation (13, 14). Functional studies identified DACH1 as a tumor suppressor in several cancer types, such as breast cancer, colorectal cancer, and renal cell carcinoma, and so on (15–17). Mechanism exploration showed that DACH1 participated in the negative regulation of cell cycle, epithelial-mesenchymal transition (EMT) and reduction of the subpopulation of cancer stem cell (CSC) (18–20). DACH1 also controlled secretion of multiple chemokine, including CXCL5, CXCL8, and IL-6 (21–23).

The chemokine (C-X-C motif) ligand 1 (CXCL1) belongs to CXC chemokine family. CXCL1 was regulated by multiple signal pathways and tumor microenvironment. For example, tumor necrosis factor (TNF) and vascular endothelial growth factor (VEGF) stimulated CXCL1 expression via JNK, p38 MAPK, and PI-3K/Akt signaling pathways in human lung carcinoma epithelial cells (24, 25). CXC receptor 2 (CXCR2) is a common receptor for CXC chemokines (26). The interaction of CXCL1 and CXCR2 promoted the development of malignant carcinoma, including proliferation, migration, angiogenesis, and therapy-resistance (27–30). Tumor-derived CXCL1 promoted the growth of lung cancer by recruiting neutrophils from peripheral blood into tumor tissues (31). The interaction between tumor cells with neutrophils increased the expression of metastasis-related genes (CXCR4, CXCR7, MMP12, MMP13, IL-6, TGF-β) (32). Consistent with the above result, the level of circulating CXCL1 in patients with metastasis was higher than patients in stage IA-IIB NSCLC (33). Besides, a study demonstrated that the expression of CXCL1 in tumor cells was elevated after paclitaxel and docetaxel treatment (34). Blocking CXCR2 enhanced chemotherapeutic response, suppressed tumor growth, angiogenesis, and metastasis (34, 35). In addition, low-dose radiation induced expression of CXC chemokines (CXCL1, CXCL2, CXCL6) in normal human fibroblasts (36).

The present study investigated the association between the expression profiles of CXCL1 and the clinicopathological features, as well as the prognostic value of CXCL1 in NSCLC. Besides, we also explored the relationship between DACH1 and CXCL1 in NSCLC.

## Materials and Methods

### Cell Culture and Transfection

Human lung cell lines (A549 and SKLU-1) were originally obtained from ATCC (Manassas, VA, USA) and we acquired from Dr. Guoan Chen in University of Michigan (37). These were cultured in RPMI-1640 medium (HyClone) with 10% fetal bovine serum (FBS) (Gibco), and HEK 293T cells were cultured in Dulbecco's modified Eagle's medium (DMEM) (HyClone) with 10% FBS. Cells were maintained at 37°C in a 5%CO2 humidified incubator. Plasmids encoding wild type DACH1 as previous description were subcloned to lentivirus expression vectors (38). DACH1 stable cell lines were characterized by inverted fluorescence microscopy and by immunofluorescence as previously described (11).

### Immunohistochemistry Staining

Two commercially available tissue microarray (TMA) slides (BC041115c, US Biomax and HLug-Ade150Sur-02, Outdo Biotech) were purchased for immunohistochemistry (IHC). BC041115c, with core diameter of 5 mm containing 40 cases of squamous cell carcinomas, 48 adenocarcinomas, 3 adenosquamous carcinomas, 4 bronchioloalveolar carcinomas, 3 large cell carcinomas, 8 small cell carcinomas, 4 lung atypical carcinoids and 10 normal lung tissues was used to detect the expression of CXCL1. HLug-Ade150Sur with 75 matched pairs of human lung adenocarcinoma and adjacent lung tissues was used to evaluate the prognostic value of CXCL1 based on the detailed survival data. In addition, the protein level of DACH1, cyclin D1, Ki67, and CXCL1 in A549-vector and A549-DACH1 tumor tissues from xenograft mice models were examined by IHC. The specific primary antibodies include anti-CXCL1-antibody (ProteinTech), anti-DACH1-antibody (ProteinTech), anti-Ki67-antibody (Abcam), and anti-cyclin D1-antibody (MAIXIN-Bio). IHC staining was performed by Bios Biotech, Inc., with a 2-step protocol as previously described (39). Slide images were captured by Mv Image software.

### Analysis and Quantification of Staining

For quantification, at least three fields at 200× magnification of each spot were selected for IHC scoring. The immunohistochemical score were assessed independently by two experienced pathologists without knowledge of patients' characteristics. Scores were calculated on intensity and percentage of positive staining tumor cells in the whole tissue stains according to the Fromowitz Standard as described (40). Briefly, the staining intensity was scored as 0 (no staining), 1 (weak staining, light yellow), 2 (moderate staining, yellow brown) and 3 (strong staining, brown). The percentage of stained tumor cells were classified as 1 (0–25% staining), 2 (26–50% staining), 2 (51–75% staining), and 3 (76–100% staining). The multiplication for intensity and percentage was utilized to represent the protein levels of CXCL1. The final score was categorized as low (scores <6) or high (scores ≥6).

### Enzyme-Linked Immunosorbent Assay (ELISA)

To evaluate the serum concentration of CXCL1, we collected the blood samples from about 100 patients who were diagnosed as ADC at various stages by April 2015 in Tongji hospital. The clinical samples collection and protocols were approved by the ethics committee of the Tongji Hospital of Huazhong University of Science and Technology. Serum samples from patients with obvious symptom of inflammation were excluded. The ELISA kit for CXCL1 was purchased from Cloud-Clone Corp. Diluted samples, including standard CXCL1 content, negative control and serums, are pipetted into 96 well plate pre-coated with anti-CXCL1 antibody. All procedures were performed following standard protocols with manual. After stopping the reaction, optical densities (OD) were measured at 450 nm using BioTek microplate reader. The intensity of this signal is directly proportional to the concentration of human CXCL1 present in the standard specimen. Standard curve demonstrated the linearity *R*^2^ = 0.9947.

### Searchlight Proteome Array

CXCL1 in cell supernatant samples was measured using SearchLight proteome arrays (Pierce Biotechnology, Woburn, MA). Briefly, the samples including A549-vector, A549-DACH1 and SKLU-1-vector, SKLU-1-DACH1 were diluted to 1:5, 1:50 or 1:1,000 before 1 h incubation on the array plate that was pre-spotted with capture antibodies specific for CXCL1. Plate was decanted and washed three times before adding a cocktail of biotinylated detection antibody to each well. After incubating with detection antibody for 30 min, plate was washed three times and incubated for 30 min with streptavidin-horseradish peroxidase. Plate was again washed before adding SuperSignal Femto Chemiluminescent substrate. The plates were immediately imaged using the SearchLight imaging system, and data was analyzed using Array Vision software.

### Human Cytokine Antibody Array

Human cytokine antibody array III kit (Ray Biotech Cat^#^ AAH-CYT-3-4) was purchased to detect the CXCL1 level in the supernatant of lung cancer cell lines. The detected samples included cell-free supernatant of A549-vector, A549-DACH1, SKLU-1-vector, and SKLU-1-DACH1. The operation steps are carried out according to the protocol provided by the manufacturer, as previously described (11).

### Meta-Analysis for CXCL1 on Published Gene Expression Omnibus Databases

We analyzed relevant Gene Expression Omnibus (GEO) databases for mRNA expression of CXCL1. We obtained these published datasets from ArrayExpress and Oncomine (41). And the included datasets must meet the following criteria: (a) the datasets were about human NSCLC cancer; (b) CXCL1 mRNA expression was measured in these databases; (c) these databases should include clinical characteristics of patients. As for datasets with the same population, the most recent or complete dataset was chosen. Finally, a total of 20 independent human NSCLC microarray databases with the mRNA expression of CXCL1 and clinical information were enrolled in this systematic analysis. The median expression of CXCL1 was used as cutoff value. overall survival (OS) and progression-free survival (PFS) were evaluated by Cox proportional hazard ratio (HR) and 95% confidence interval (95% CI). HR >1 indicated that high expression of CXCL1 predicted worse survival of patients. The meta-analysis was performed by the STATA software (Stata Corp LP, College Station, TX, USA). And statistical analysis was performed based on the guidelines of Meta-Analysis of Observational Studies.

### Kaplan–Meier Plotter

Kaplan–Meier survival curves with HR and log-rank *P*-value were calculated and plotted on the online Kaplan–Meier plotter (http://kmplot.com/analysis). The background database, required from GEO, offers gene expression data and survival information. The Affymetrix ID for CXCL1 was 204470_at, which includes 2,435 cases of lung ADC and SQC. The follow up time was threshold at 150 months. The patients were divided by median expression of CXCL1. The Kaplan–Meier survival curves were downloaded from the website. And the blend curves were obtained from IBM SPSS Statistics 19.0 based on GSE31210. All curves were resized in Adobe Illustrator CS6.

### Statistical Analysis

Statistical analysis between groups were calculated by Student's *t-*test and one-way ANOVA and the significance level was set at 0.05. Univariate cumulative survival analyses for PFS and OS were calculated using Kaplan–Meier method with the log-rank test. The correlation analysis was calculated according to Person χ^2^-test. Statistical analyses were conducted using SPSS 19.0 and GraphPad Prism 6.0. All data were presented as the mean ± SD.

## Results

### Upregulation of CXCL1 Protein in Various Kinds of Lung Cancer

**Figure 1:**
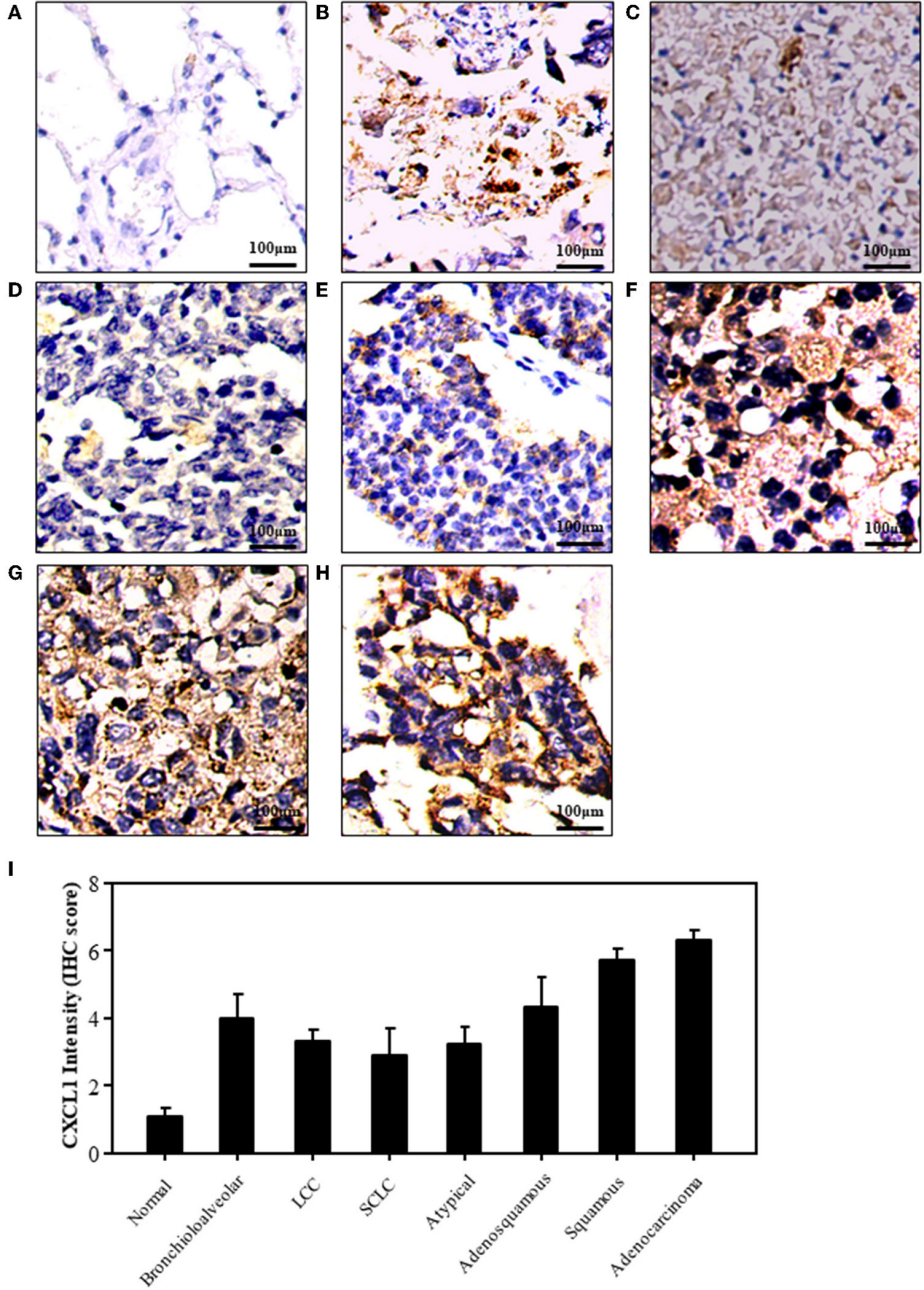
The expression of CXCL1 protein in lung cancers. **(A)** Normal tissue **(B)** Bronchioloalveolar cancer **(C)** lung large cell cancer (LCC) **(D)** small cell lung cancer (SCLC) **(E)** Atypical lung cancer **(F)** Adenosquamous cancer **(G)** Squamous cell carcinoma **(H)**. Lung adenocarcinoma **(I)** CXCL1 IHC score.

We detected the expression of CXCL1 in normal and lung cancer tissues by IHC staining. The representative images of normal and cancer tissues were shown in Figures 1A–H. The results indicated that the expression of CXCL1 in cancer tissues was apparently higher than that of normal tissue. Among these kinds of lung cancers, the CXCL1 expression in adenocarcinoma was the highest (Figure 1I). However, the CXCL1 expressed in SCLC was significantly less than that of NSCLC. The average scores of normal, SCLC, adenocarcinoma and squamous cell carcinoma were 1.1 ± 0.23, 2.9 ± 0.81, 6.3 ± 0.27, and 5.7 ± 0.31, respectively.

### Expression of CXCL1 Correlated With Progression and Prognosis of Lung ADC

**Figure 2:**
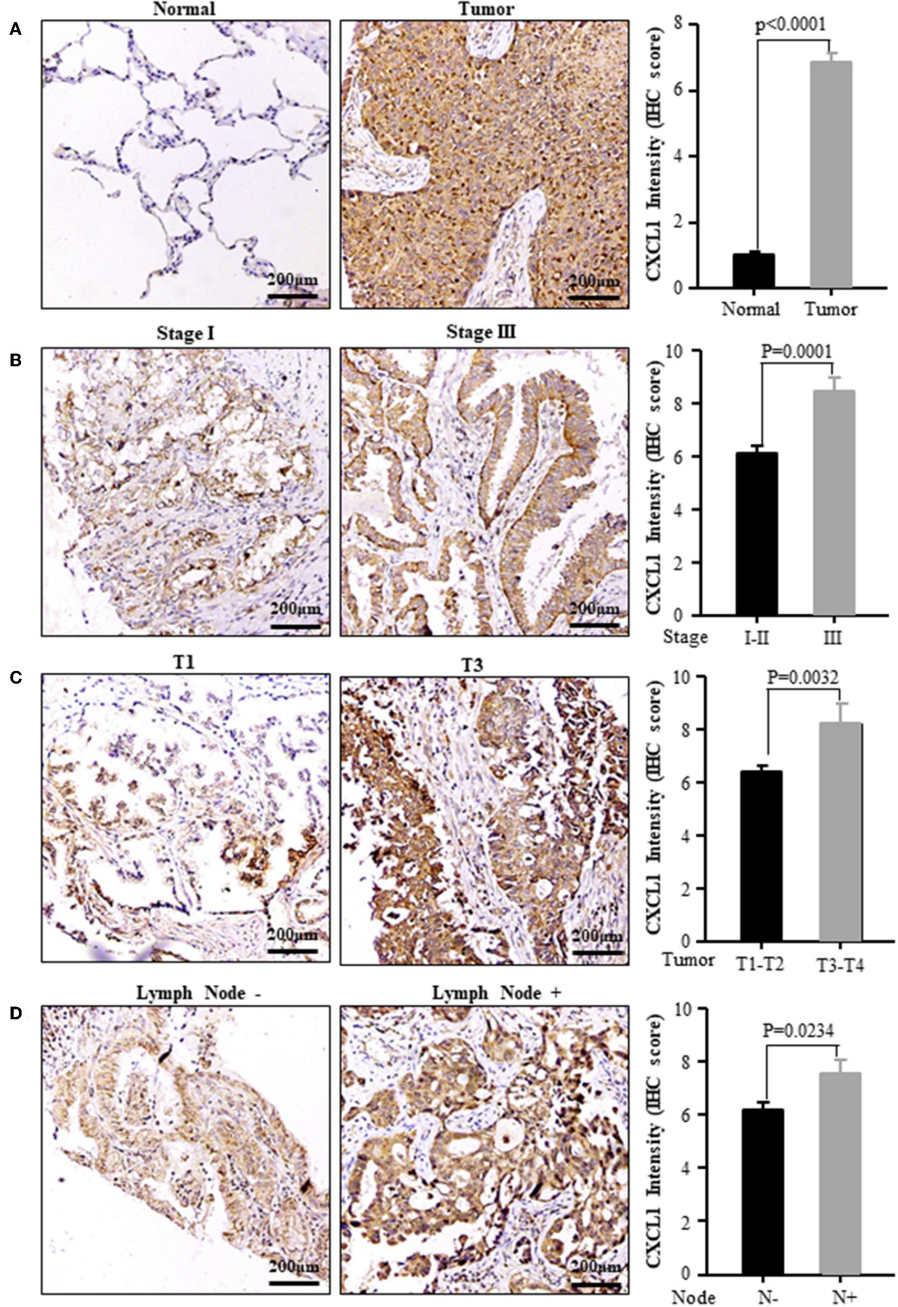
The expression of CXCL1 protein was positively correlated with the progression of ADC patients. **(A)** Normal vs. Tumor **(B)** TNM stage I vs. III **(C)** Tumor size T1 vs. T3 **(D)** Lymph node metastasis positive vs. negative. Left panel: representative images, right panel: CXCL1 IHC score.

**Figure 3:**
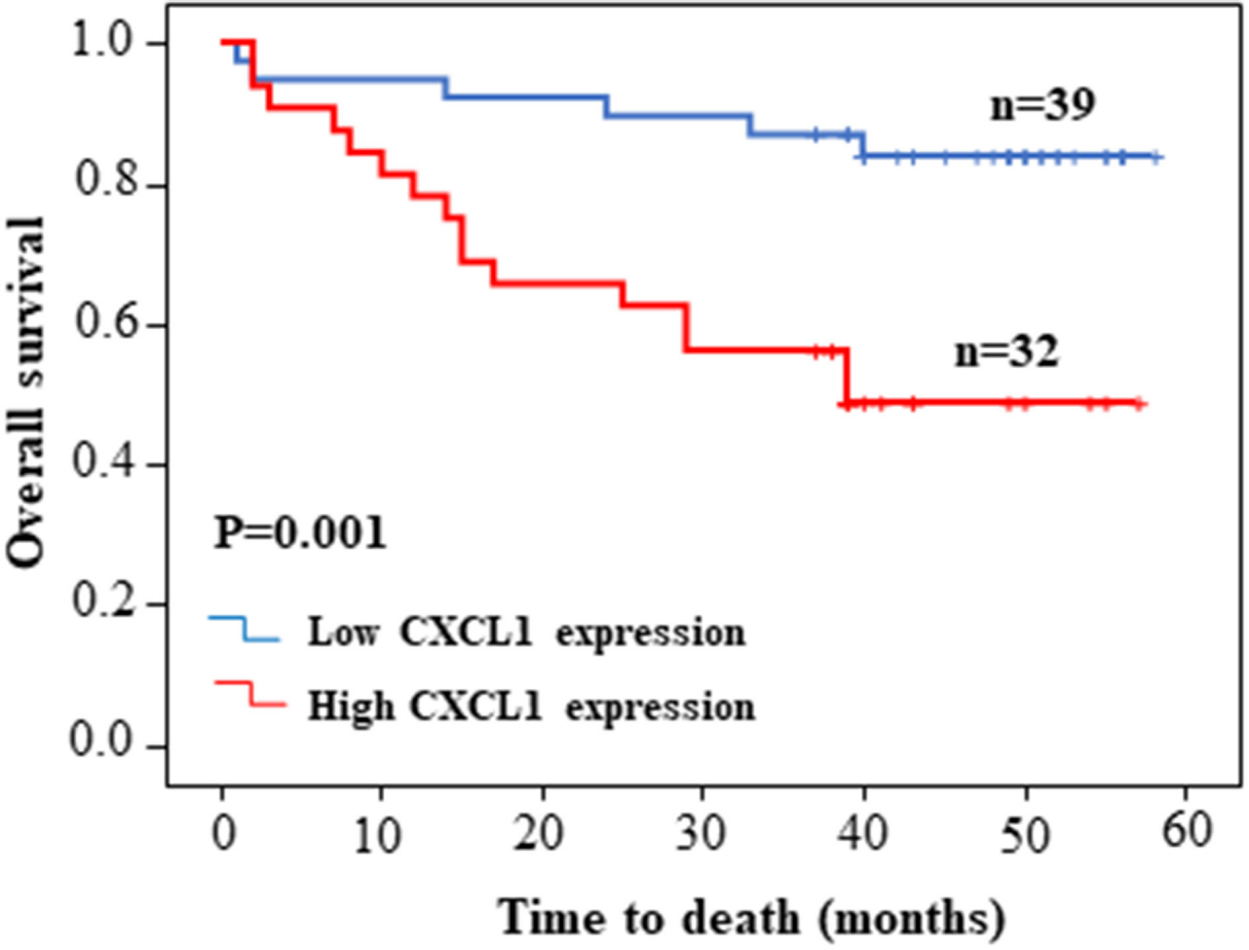
Kaplan–Meier survival curve of patients with low or high CXCL1 expression based on the survival data in tissue microarray slide.

Considering the high protein abundance of CXCL1 in lung adenocarcinoma, we further analyzed the expression of CXCL1 in a TMA containing lung adenocarcinoma with matched adjacent lung tissue and clinicopathological parameters. Representative images of IHC staining for adjacent tissues and adenocarcinoma were shown in Figure 2. In comparison with adjacent lung tissues, the CXCL1 protein in lung adenocarcinoma was evidently elevated (*P* < 0.0001) (Figure 2A). The protein abundance of CXCL1 was positively correlated with tumor TNM stage (*P* = 0.0001), tumor size (*P* = 0.0032) and lymph node metastasis (*P* = 0.0234) (Figures 2B–D). However, there was no statistical difference with grade. According to the average score of staining, CXCL1 expression was divided into CXCL1 high (*n* = 32) and low (*n* = 39) subgroups. Medium OS time of the CXCL1 high and low subgroups were 30 ± 2.98 and 48 ± 2.14 months, respectively, which indicated that they had significant difference of survival (Kaplan–Meier log-rank test, *P* = 0.001, Figure 3). We also explored the relationship between CXCL1 expression and clinicopathological features of 71 ADC patients. As shown in Table 1, CXCL1 expression was only correlated with TNM stage. Besides, we investigated the correlation between cumulative OS and clinicopathological parameters by univariate Cox regression analysis including age, sex, grade, tumor size, lymph node metastasis, TNM stage and CXCL1 expression (Table 2). The results demonstrated that TNM stage (HR = 7.393; 95% CI 1.267–43.135; *p* = 0.026) and CXCL1 expression (HR = 3.533; 95% CI 1.232–10.132; *p* = 0.019) were prognostic factors for OS. Multi-various Cox analysis revealed that TNM stage (HR = 4.499; 95% CI 1.853–10.919; *p* = 0.001) and CXCL1 expression (HR = 2.916; 95% CI 1.099–7.739; *p* = 0.032) were independent prognostic factors for OS.

**Table 1:** Correlations between CXCL1 expression and clinicopathological features of 71 ADC patients.

**Variable**	**N**	**CXCL1 IHC score**		***P*-value**
		**≤6 (low expression)**	**>6 (high expression)**	
**Age**				0.424
≤60	37	22	15	
>60	34	17	17	
**Sex**				0.267
Male	37	18	19	
Female	34	21	13	
**Tumor size**				0.191
T1-T2	56	33	23	
T3-T4	15	6	9	
**TNM stage**				0.033
I–II	53	33	20	
III	18	6	12	
**Lymph node metastasis**				0.110
N–	33	21	12	
N+	36	16	20	
**Grade**				0.839
I, I–II	13	8	5	
II	42	22	20	
II–III, III	16	9	7	

**Table 2:** The expression of CXCL1 is an independent prognostic factor for ADC patients.

**Variables**	**Univariate analysis**		**Variable selection**	
	**HR (95%CI)**	***P*-value**	**HR (95% CI)**	***P*-value**
Age (≤60 = 0, >60 = 1)	1.242 (0.516–2.993)	0.629		
Sex (Male vs. Female)	1.136 (0.381–3.383)	0.819		
Grade (I, I-II/II/II-III, III)	1.397 (0.597–3.272)	0.441		
tumor size (T1-2/T3-4)	1.092 (0.348–3.427)	0.880		
Lymph node metastasis (N–/N+)	0.530 (0.099–2.844)	0.459		
Stage (I–II/III)	7.393 (1.267–43.135)	0.026	4.499 (1.853–10.919)	0.001
CXCL1 expression (≤6/>6)	3.533 (1.232–10.132)	0.019	2.916 (1.099–7.739)	0.032

### CXCL1 Protein Increased in the Serum of Patients With Lung ADC

**Figure 4:**
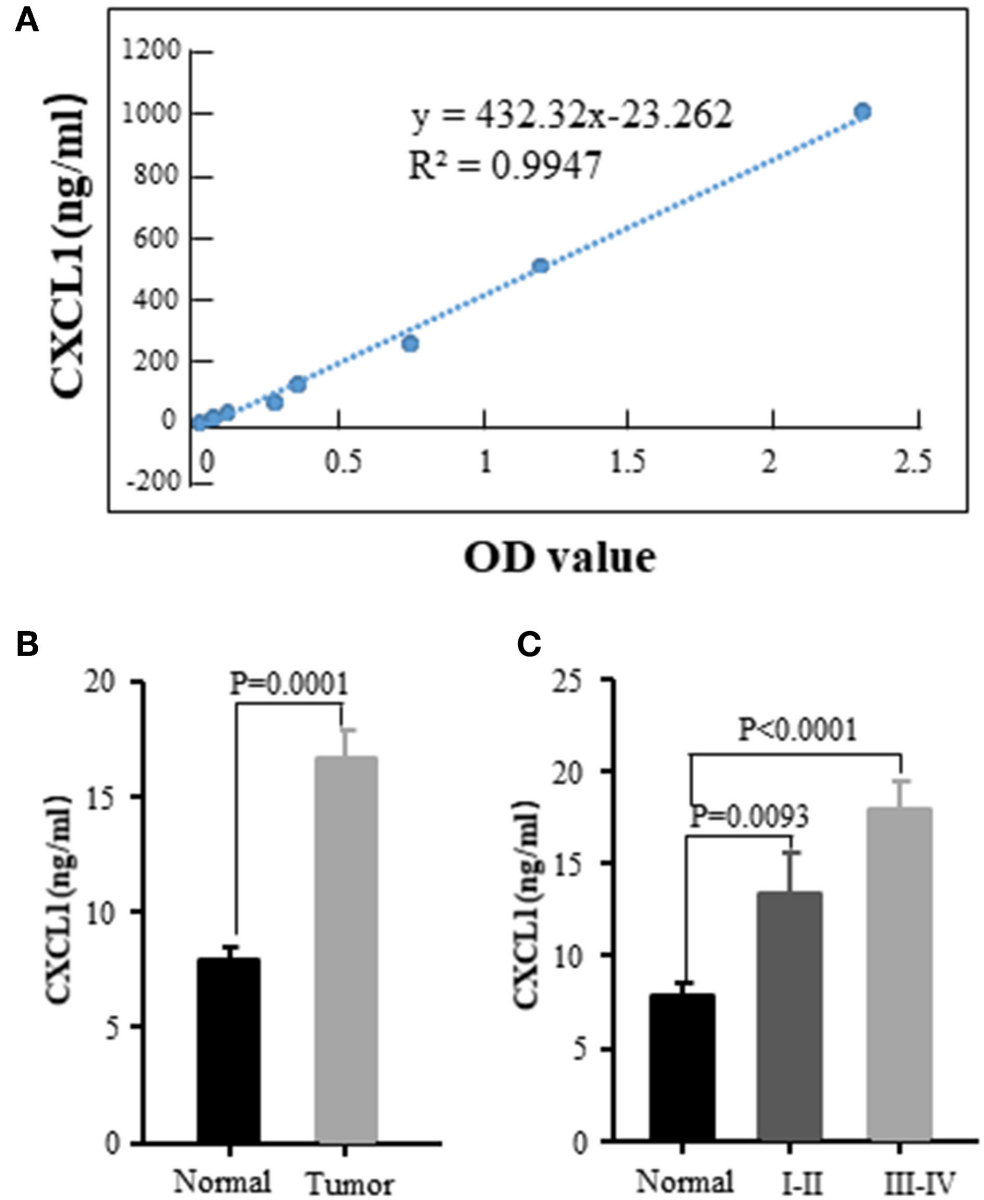
Elevated CXCL1 protein in serum of patients with ADC. **(A)** Standard curve of CXCL1 with OD value **(B)** Serum CXCL1 in healthy donors and lung ADC patients **(C)** Serum CXCL1 protein was positively correlated with TNM stage.

We also examined the serum CXCL1 concentration by commercial ELISA kit. The standard curve between CXCL1 amount and OD value was excellent linear with *R*^2^ = 0.9947 (Figure 4A). The tested serum samples were from 20 normal donors and 56 ADC patients with various tumor stages and histological grades. Sixteen of fifty-six patients were in early stage (I-II) and the rest were in III-IV stage. The results confirmed that CXCL1 expression in serum was considerably elevated in adenocarcinoma compared with normal samples (*P* = 0.0001, Figure 4B). High expression of CXCL1 tended to be associated with tumor progression, but it did not reach the statistical significance (Figure 4C).

### High Level of mRNA CXCL1 Predicted Progression and Worse Clinical Outcomes for Patients With NSCLC

**Figure 5:**
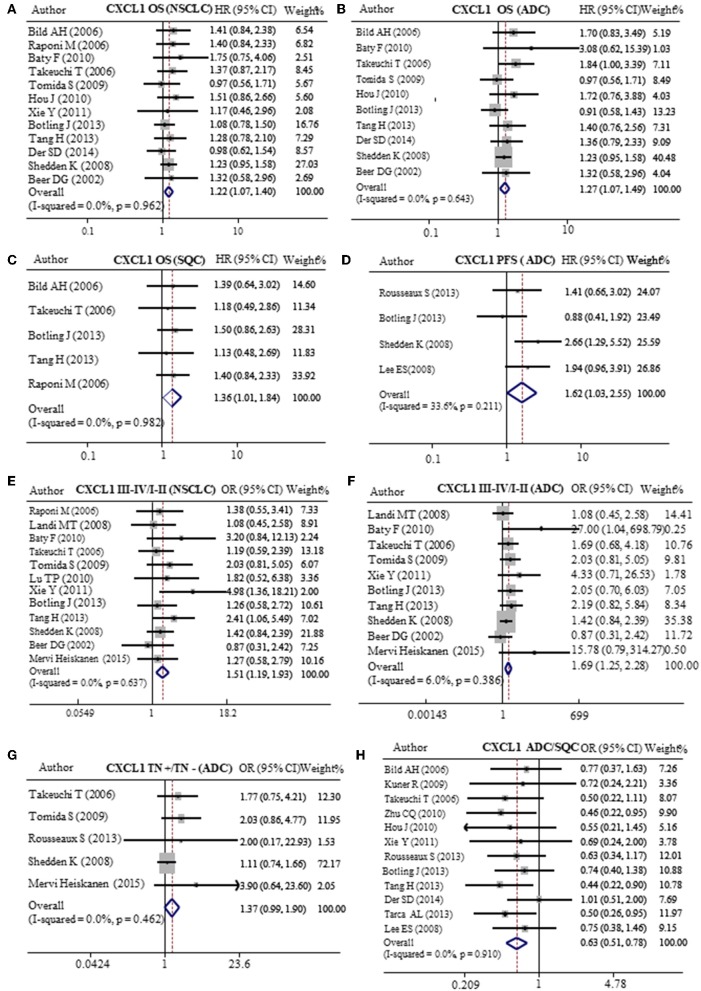
Meta-analysis of mRNA CXCL1 expression in lung cancers. Relative risk of CXCL1 mRNA expression to OS in NSCLC **(A)**, ADC **(B)**, and SQC **(C)**. **(D)** Relative risk of CXCL1 mRNA expression to PFS in ADC. The forest plot of relative mRNA expression of CXCL1 between III–IV and I–II patients in NSCLC **(E)** and ADC **(F)**. **(G)** The forest plot of relative mRNA expression of CXCL1 between lymph node metastasis positive and negative patients in ADC **(H)** OR value of CXCL5 in ADC vs. SQC.

In order to verify the prognostic value of CXCL1 in lung cancer, we analyzed 20 published GEO databases (summarized in Table 3) mainly containing NSCLC patients and corresponding clinicopathological parameters. The patients were divided into CXCL1 high and low based on the median CXCL1 mRNA value. Our results of meta-analysis indicated that higher expression of mRNA CXCL1 was strongly correlation with worse OS whether it is in NSCLC (HR: 1.22, 95%CI: 1.07–1.40, *P* = 0.962, and *I*^2^ = 0.0%) (Figure 5A) or ADC (HR: 1.27, 95%CI: 1.07–1.49, *P* = 0.643, and *I*^2^ = 0.0%) (Figure 5B) or SQC (HR: 1.36, 95%CI: 1.01–1.84, *P* = 0.982, and *I*^2^ = 0.0%) (Figure 5C). However, only in ADC, the high mRNA expression of CXCL1 contributed to shorter PFS (HR: 1.62, 95%CI: 1.03–2.55, *P* = 0.211, and *I*^2^ = 33.6%) (Figure 5D). Furthermore, we found that mRNA CXCL1 was increased in TNM stage of III-IV in NSCLC (OR: 1.51, 95%CI:1.19–1.93, *P* = 0.637, and *I*^2^ = 0.0%) (Figure 5E) and ADC (OR: 1.69, 95%CI:1.25–2.28, *P* = 0.386, and *I*^2^ = 6.0%) (Figure 5F), but not in SQC. Similarly, mRNA expression of CXCL1 was elevated in ADC patients with lymph node metastasis (OR: 1.37, 95%CI: 0.99–1.90, *P* = 0.462, and *I*^2^ = 0.0%) (Figure 5G). There was no statistical significance between CXCL1 mRNA level and histological grade. The mRNA expression of CXCL1 in SQC was higher than that in ADC (Figure 5H).

**Table 3:** Characteristics of studies included for meta-analysis.

**First author**	**Accession number**	**Year**	**Duration (months)**	**Patients number**	**Histology**	**Detection**	**Platform**
Bild et al. (42)	GSE3141	2006	88	111	NSCLC	Microarray	GPL570
Raponi et al. (43)	GSE4573	2006	144	129	SQC	Microarray	GPL570
Baty et al. (44)	GSE11117	2010	44	56	NSCLC	Microarray	GPL6650
Takeuchi et al. (45)	GSE11969	2006	108	149	NSCLC	Microarray	GPL7015
Tomida et al. (46)	GSE13213	2009	109.8	117	ADC	Microarray	GPL6480
Hou et al. (47)	GSE19188	2010	130	91	NSCLC	Microarray	GPL570
Xie et al. (48)	GSE29013	2011	82	55	NSCLC	Microarray	GPL570
Botling et al. (49)	GSE37745	2013	120	196	NSCLC	Microarray	GPL570
Tang et al. (50)	GSE42127	2013	120	176	NSCLC	Microarray	GPL6884
Der et al. (51)	GSE50081	2014	60	181	NSCLC	Microarray	GPL570
Shedden et al. (52)	GSE68465	2008	204	442	ADC	Microarray	GPL96
Beer et al. (53)	GSE68571	2002	110.6	86	ADC	Microarray	GPL80
Rousseaux et al. (54)	GSE30219	2013	256	293	NSCLC	Microarray	GPL570
Lee et al. (55)	GSE8894	2008	120	138	NSCLC	Microarray	GPL570
Landi et al. (56)	GSE10072	2008	68.4	74	ADC	Microarray	GPL96
Lu et al. (57)	GSE19804	2010	NR	60	ADC	Microarray	GPL570
Kuner et al. (58)	GSE10245	2009	70.6	58	NSCLC	Microarray	GPL570
Zhu et al. (59)	GSE14814	2010	108	133	NSCLC	Microarray	GPL96
Tarca et al. (60)	GSE43580	2013	NR	150	NSCLC	Microarray	GPL570
Heiskanen et al. (61)	GSE68787	2015	NR	35	ADC	Microarray	GPL20187

*Cut-off value: median expression. NR, not report; NSCLC, non-small-cell lung cancer; ADC, lung adenocarcinoma; SQC, lung squamous cell carcinoma*.

### DACH1 Reduced the Expression of CXCL1 *in vitro*

**Figure 6:**
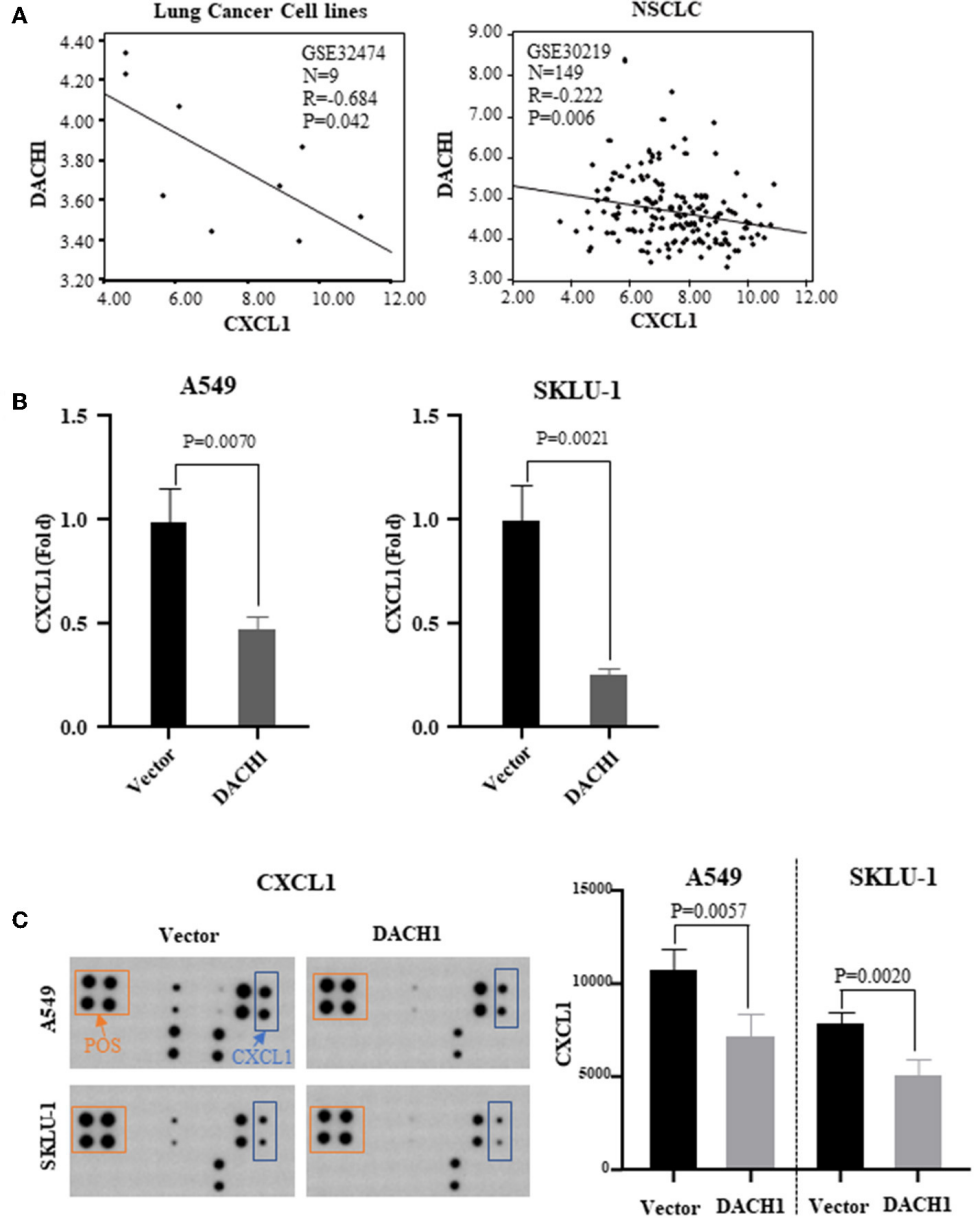
Correlation between CXCL1 and DACH1. **(A)** CXCL1 negatively correlated with DACH1 in mRNA level based on published databases. SearchLight proteome array **(B)** and human cytokine array III **(C)** demonstrated that CXCL1 protein was suppressed by DACH1 both in A549 and SKLU-1 cell lines.

Our previous study has demonstrated that DACH1 was a tumor suppressor factor and negatively correlated with CXCL5 (22). And CXCL5 contributed to the tumorigenesis and poor prognosis in NSCLC (62). Both CXCL1 and CXCL5 belong to CXC chemokine family. Therefore, we analyzed lung cancer cell line dataset (GSE32474) and tumor tissue dataset (GSE30219) and found that CXCL1 inversely correlated with DACH1 (Figure 6A, *R* = 0.684, *P* = 0.042; *R* = 0.222, *P* = 0.006). To test whether DACH1 directly regulated CXCL1, two lung cancer cell lines A549 and SKLU-1 were transferred with control or DACH1 plasmid, respectively. We collected the supernatant of A549-vector, A549-DACH1, SKLU-1-vector and SKLU-1-DACH1 cell lines and detected the protein level of CXCL1 by SearchLight proteome array. The results demonstrated that DACH1 significantly reduced the expression of CXCL1 in A549-DACH1 and SKLU-1-DACH1 cells (Figure 6B). In addition, the human cytokine array III also proved that CXCL1 was indeed suppressed by DACH1 both in A549 and SKLU-1 cells. (Figure 6C).

### DACH1 Reduced the Expression of CXCL1, Cyclin D1, and Ki67 *in vivo*

**Figure 7:**
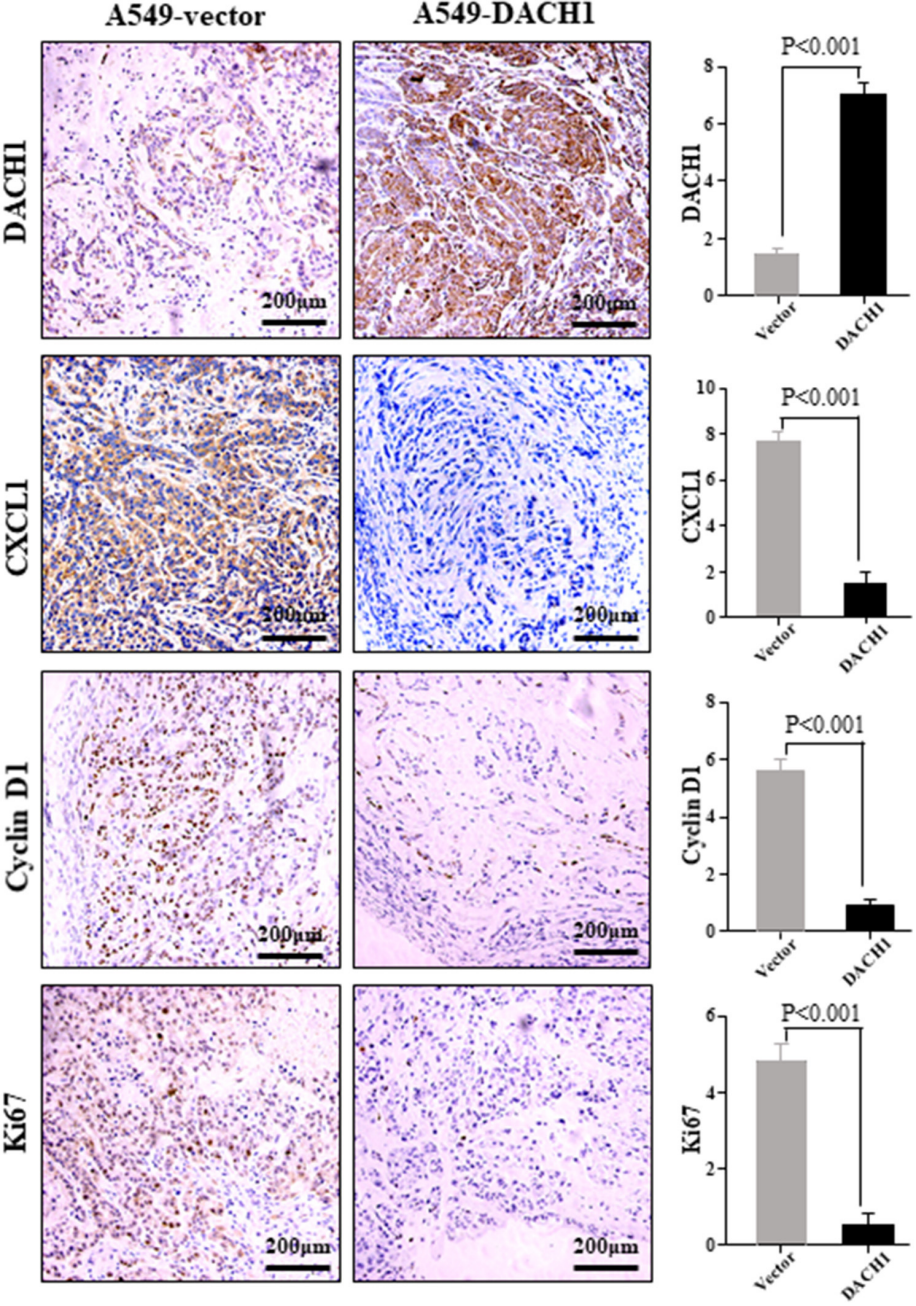
Representative images of DACH1, CXCL1, cyclin D1, and Ki67 IHC staining from xenograft tumor tissues subcutaneously implanted with A549-vector and A549-DACH1 cells.

Previous study showed that ectopic expression of DACH1 inhibited tumor growth (11). IHC staining was performed to evaluate the protein level of DACH1, CXCL1, cyclin D1, and Ki67 in nude mice xenograft tumor tissues from cells expressing vector control and DACH1. Representative images of IHC staining and scoring results for DACH1, CXCL1, cyclin D1, and Ki67 were shown in Figure 7. Our results demonstrated that overexpression of DACH1 (*P* < 0.001) obviously decreased the expression of CXCL1 (*P* < 0.001), cyclin D1 (*P* < 0.001), and Ki67 (*P* < 0.001), suggesting that DACH1 not only reduced the expression of CXCL1, but also inhibited proliferation biomarkers cyclin D1 and Ki67 *in vivo* at protein level.

### High CXCL1 and Low DACH1 Expression Predicted Poor Survival in ADC

**Figure 8:**
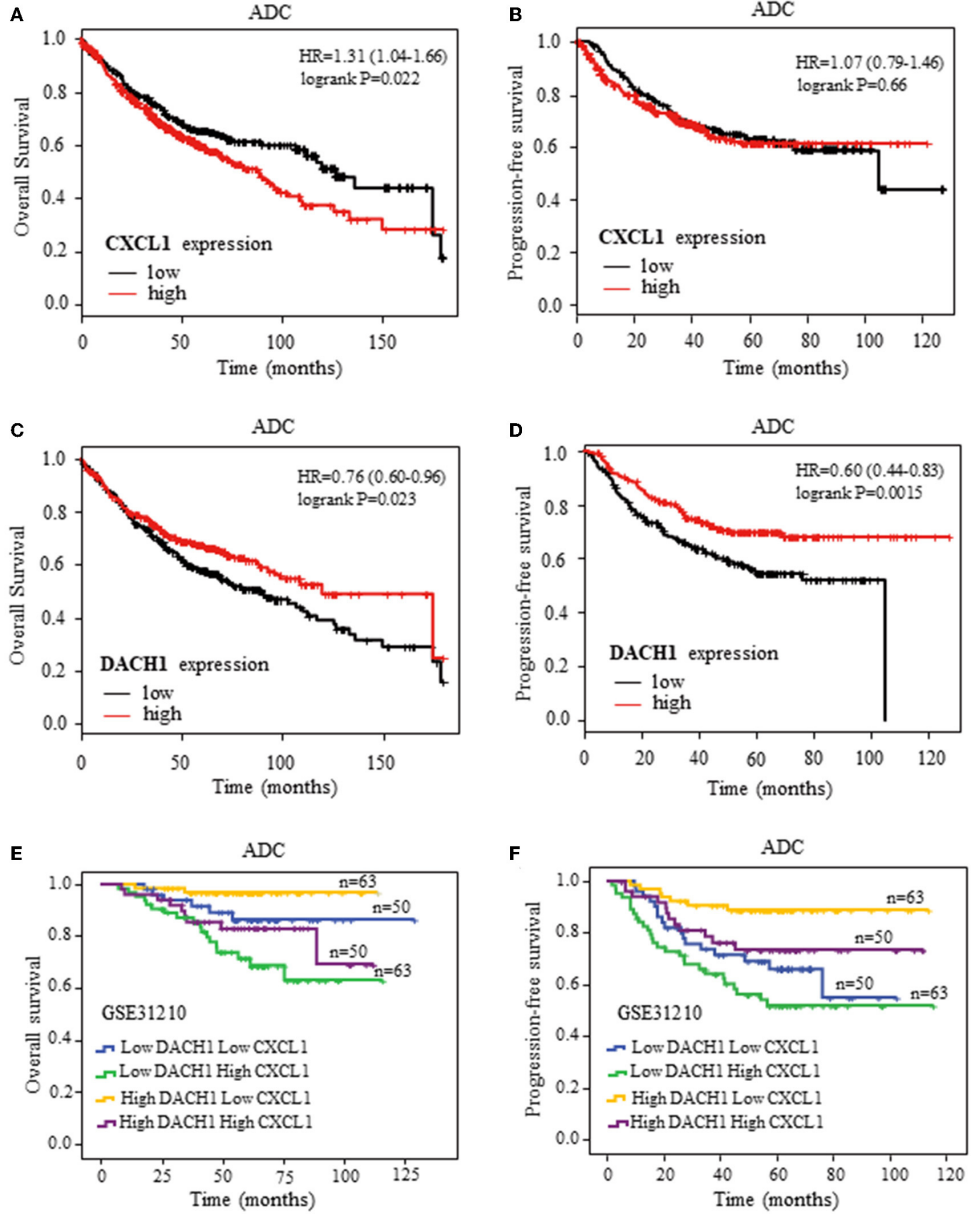
Kaplan–Meier survival curves. Survival curves of CXCL1 with OS **(A)** and PFS **(B)** for patients with ADC. Survival curves of DACH1 with OS **(C)** and PFS **(D)** for patients with ADC. The blend Kaplan–Meier survival curves of CXCL1 and DACH1 with OS **(E)** and PFS **(F)** for patients with ADC in GSE31210.

To further confirm the clinical significance of CXCL1 and DACH1expressions, we first investigated the prognostic value of CXCL1 and DACH1 for patients with ADC on kmplot.com, respectively. The Kaplan–Meier curves from univariate Cox regression showed that patients with higher mRNA expression of CXCL1 tended to have shorter median OS (HR:1.31, 95%CI:1.04–1.66, *P* = 0.022) (Figure 8A). Nevertheless, this tendency was not observed in PFS (HR:1.07, 95%CI:0.79–1.46, *P* = 0.66) (Figure 8B). As expected, higher mRNA expression of DACH1 was significantly associated with better OS (HR:0.76, 95%CI:0.60–0.96, *P* = 0.023) (Figure 8C) and PFS (HR:0.60, 95%CI:0.44–0.83, *P* = 0.0015) (Figure 8D). In order to obtain more precise prognostic information for ADC patients, we performed combination analysis for the expression of CXCL1 and DACH1 in published database GSE31210. Apparently, patients characterized by high DACH1 with low CXCL1 achieved longest median OS (Figure 8E) and PFS (Figure 8F). The results proved that DACH1 was a potential prognostic factor for better clinical outcomes, while CXCL1 was an adverse biomarker for ADC patients. Combined detection of CXCL1 and DCCH1 provided more precise information for prognosis.

## Discussion

Accumulating evidence has proved that CXCL1 plays an important role in the development of various malignant tumors. CXCL1, as an inflammatory factor, participates in the recruitment of granulocytes (63). Tumor-derived CXCL1 contributes to tumor-associated neutrophils infiltration in lung cancer which promotes tumor growth (31). Colorectal CSCs secrete CXCL1 and CXCL2 to attract neutrophils, which promoted tumorigenesis of colorectal cancer cells via interleukin-1β (64). CXCL1/2 recruit CD11b(+)Gr1(+) myeloid cells into the tumor, which produce chemokines including S100A8/9 that enhance cancer cell survival, chemoresistance and metastasis (28). Feedback loop between NF-κB and CXCL1/-2 enhanced metastasis formation *in vivo* (65). In ER-negative breast cancer, CXCL1 is highly expressed and stimulates cancer cell migration and invasion via activating the ERK-MMP2/9 signaling pathway (66).

Through immunohistochemical analysis, we found that CXCL1 protein expressed in ADC was higher than in other kinds of lung cancer. Therefore, we further evaluated the expression level of CXCL1 in ADC and its relationship with clinicopathological characteristics and prognosis. The analysis showed that the expression of CXCL1 in ADC was positively related to the TNM stage, tumor size and lymph node metastasis. CXCL1 was also positively associated with tumor grade, though it did not reach statistical significance. The survival curve plotted with survival information exhibited that the higher CXCL1 level accompanied with the shorter survival time. Previous study reported that circulating CXCL1 combining CCL18 could be as tumor markers for the differential diagnosis between ovarian cancer and benign ovarian masses (67). Therefore, except for tumor tissues, we also collected the peripheral blood of ADC patients and normal donors to detect the amount of circulating CXCL1 by ELISA. The results demonstrated that the serum CXCL1 of ADC patients obviously higher than that of normal donors and correlated with TNM stage though there is no statistical significance, possibly because of the limited samples. Our results were in agreement with previous study that NSCLC patients with metastasis had high levels of CXCL1 than patients in stage IA-IIB (33). From the above results, we speculate that CXCL1 could be used as an indicator for monitoring cancer progression. In particular, detection of circulating CXCL1 would be a more convenient and acceptable method.

The analysis results of 20 databases showed that mRNA CXCL1 positively associated with OS, PFS, TNM stage and lymph node metastasis for the ADC patients. But for the SQC patients, higher mRNA CXCL1 only related with shorter OS. Therefore, although the mRNA level of CXCL1 in SQC is higher than that of in ADC, the functions of CXCL1 were more obvious in ADC, suggesting that CXCL1 protein abundance and functions were also regulated by other factors.

As previous studies proved that DACH1 was considered as a tumor suppressor in several kinds of cancers including lung cancer and regulated multiple cytokine expression (8, 11, 14). Therefore, we performed the correlation analysis between CXCL1 and DACH1 in lung cancer cell lines and cancer tissues. The results suggested that CXCL1 negatively correlated with DACH1 at mRNA level. *In vitro* study confirmed that secretion of CXCL1 protein was significantly suppressed by DACH1 in lung cancer cell lines A549 and SKLU-1. Our previous study has demonstrated that DACH1 overexpression remarkably inhibited the growth of lung cancer in nude mice (11). The tumor tissues isolated from those nude mice were further immunized with four antibodies: DACH1, CXCL1, cyclin D1, Ki67. The results showed that DACH1 significantly inhibited the expression of CXCL1, cyclin D1, and Ki67 *in vivo*. Previous finding demonstrated that tumor derived CXCL1 promoted the growth of lung cancer (31), our study suggested that the tumor suppress function of DACH1 might be through inhibiting CXCL1. CXCL1 was regulated by several pathways, including JNK, p38 MAPK, and PI-3K/Akt signaling pathways (24, 25). Our previous study indicated DACH1 suppressed CXCL8 through AP-1 and NF-κB sites of CXCL8 promoter depending on the DS domain of DACH1 in NSCLC (11). And other study showed that DACH1 repressed CXCL6 and CXCL8 in a dose-dependent manner, and repression required the DS domain in prostate cancer (21). CXCL1, CXCL6, and CXCL8 all belong to CXC chemokine family. Therefore, it is rationally assumed that CXCL1 was suppressed by DACH1 in a similar way.

Our previous study showed that DACH1 antagonized the function of CXCL5 and revealed opposite effect on prognosis (22). To explore the whether CXCL1 and DACH1 had similar role, we plotted the survival curves of CXCL1 and DACH1 for ADC patients. The results showed that high CXCL1 associated with short survival time, while as the high DACH1 predicted long OS and PFS as previously reported (22). To explore the combined effects of CXCL1 and DACH1 on prognosis, the blend curves was performed on public database GSE31210 which includes 226 samples from ADC patients. The results suggested that high CXCL1 with low DACH1 indicated worse OS and PFS. Therefore, CXCL1 combining DACH1 could more precisely stratify patients with lung ADC in term of prognosis.

## Data Availability Statement

All datasets generated for this study are included in the article/Supplementary Material.

## Ethics Statement

The studies involving human participants were reviewed and approved by the ethics committee of the Tongji Hospital of Huazhong University of Science and Technology. The patients/participants provided their written informed consent to participate in this study.

## Author Contributions

SY performed the experiments and drafted the manuscript. LX, MY, and SQ prepared the figures and tables. AL and KW designed the study and revised the manuscript. All authors contributed to this manuscript, read, and approved the final manuscript.

### Conflict of Interest

The authors declare that the research was conducted in the absence of any commercial or financial relationships that could be construed as a potential conflict of interest.

## Abbreviations

SCLC: small cell lung cancer
NSCLC: non-small cell lung cancer
ADC: adenocarcinoma
SQC: squamous cell carcinoma
EGFR: epidermal growth factor receptor
ALK: anaplastic lymphoma kinase
CXCL1: chemokine (C-X-C motif) ligand 1
CXCR2: CXC receptor 2
VEGF: vascular endothelial growth factor
TNF: tumor necrosis factor
MPE: malignant pleural effusion
DACH1: Dachshund homolog 1
RDGN: retinal determination gene network
EMT: epithelial-mesenchymal transition
ELISA: enzyme-linked immunosorbent assay
CSC: cancer stem cell
TMA: tissue microarray
OD: optical densities
GEO: Gene Expression Omnibus
OS: overall survival
PFS: progression-free survival
HR: hazard ratio.
